# Supplementary material for: Synthesis and Properties of Hydrophilic and Hydrophobic Deep Eutectic Solvents via Heating-Stirring and Ultrasound
Source: Molecules. 2024 Jun 28;29(13):3089. doi: 10.3390/molecules29133089 (PMC11243031; doi:10.3390/molecules29133089)
Supplement: Supplementary file 1 [file molecules-29-03089-s001.zip › molecules-3015289-supplementary.pdf]

Synthesis and properties of hydrophilic and hydrophobic Deep Eutectic Solvents by different methods

María Isabel Martín[1*], Irene García-Díaz [1], María Lourdes Rodríguez[1], María Concepción Gutiérrez [2], Francisco del Monte [2], Félix A. López[1*]

Supplementary material

Table S1. Bibliographic densities obtained for 25ºC for hydrophilic and hydrophobic DESs.

Hydrophilic DES	Density (g/cm^3)	Reference
ChCl:EG (1:2)	1.12	[52]
ChCl:EG (1:3)	1.12	[44]
ChCl:EG (1:4)	1.11	[46]
ChCl:U (1:2)	1.21	[53]
ChCl:U (1:2)	1.25	[43]
ChCl:Ox (1:1)	1.15	[12]
Hydrophofic DES		
Aliq:LMet (3:7)	0.88	[34]
Lid:Ac.Dec (1:2)	0.89	[54]
